# Comparative studies of alignment, alignment-free and SVM based approaches for predicting the hosts of viruses based on viral sequences

**DOI:** 10.1038/s41598-018-28308-x

**Published:** 2018-07-03

**Authors:** Han Li, Fengzhu Sun

**Affiliations:** 1 Molecular and Computational Biology Program, Department of Biological Sciences, University of Southern California, Los Angeles, CA 90089 USA; 2 Centre for Computational Systems Biology, School of Mathematical Sciences, Fudan University, Shanghai, 200433 China

## Abstract

Predicting the hosts of newly discovered viruses is important for pandemic surveillance of infectious diseases. We investigated the use of alignment-based and alignment-free methods and support vector machine using mononucleotide frequency and dinucleotide bias to predict the hosts of viruses, and applied these approaches to three datasets: rabies virus, coronavirus, and influenza A virus. For coronavirus, we used the spike gene sequences, while for rabies and influenza A viruses, we used the more conserved nucleoprotein gene sequences. We compared the three methods under different scenarios and showed that their performances are highly correlated with the variability of sequences and sample size. For conserved genes like the nucleoprotein gene, longer *k*-mers than mono- and dinucleotides are needed to better distinguish the sequences. We also showed that both alignment-based and alignment-free methods can accurately predict the hosts of viruses. When alignment is difficult to achieve or highly time-consuming, alignment-free methods can be a promising substitute to predict the hosts of new viruses.

## Introduction

Viruses are ubiquitous and can reproduce and evolve very fast. Virus infections in human can cause various diseases and are a big threat to human health. Many infectious disease studies showed that virus cross-species transmissions are highly prevalent resulting in emerging infectious diseases (EIDs)^1^. EIDs continue to pose significant public health problems as shown by the recent outbreaks of West Nile virus, SARS, MARS, and H1N1^2^. Rapidly identifying the reservoir of the new pathogenic bacterial or viral origins responsible for these diseases will help the containment, control, and prevention of the outbreaks^1,3,4^. Further, investigating the potential host of a virus can throw light on the evolutionary history of the virus, thus provide guidance on how to cut off the transmission path. The biological presumption for most of the host identification methods is that the more similar two viruses’ DNA/RNA sequences are, they are more likely to share the same host^5^.

With the availability of various databases containing different types of pathogenic microbial species, one of the most commonly used approaches for identifying the origin of the new pathogen responsible for an EID is to find similar sequences in the pathogen databases using alignment by the Smith-Waterman algorithm^6^, BLAST^7^, or other alignment tools.

Recently, several alignment-free methods have been developed for the identification of the hosts of pathogenic species. Kapoor *et al*.^8^ used relative dinucleotide frequencies and discriminant analysis to infer the hosts of novel picorna-like viruses. Aguas and Ferguson^9^ developed a feature selection method and used random forests (RF) based on the diverged nucleotide or amino acid bases among a set of aligned molecular sequences to predict the host species of pathogens. Tang *et al*.^10^ developed a support vector machine (SVM) based method using mono- and dinucleotide frequencies as features to detect the original hosts of coronaviruses with high accuracy. Kargarfard *et al*.^11^ predicted the host range of the influenza virus using various machine learning approaches. Several new alignment-free statistics including ${d}_{2}^{\ast }$ and ${d}_{2}^{S}$ for molecular sequence comparison using *k*-mers (*k*-grams, words, etc.) were developed recently^12,13^. It was shown that such measures are highly associated with the evolutionary distances estimated from alignment-based methods, thus validating the usefulness of alignment-free methods for the comparison of molecular sequences^14,15^. In this study, we investigate the effectiveness of alignment, alignment-free and machine learning based methods for inferring the hosts of viruses responsible for emerging infectious diseases.

## Results

We initially calculated the prediction accuracies of the *K*-nearest neighbors (KNN) algorithm based on the alignment method and the alignment-free distance/dissimilarity measures for *k*-mer length from 3 to 6 and the number of neighbors *K* from 1 to 10. The results for the rabies virus, coronavirus, and influenza A virus datasets are given as Figs S1, S2 and S3 in the supplementary material, respectively. These figures show that except for the Chebyshev divergence, all the other alignment-free distance/dissimilarity measures have similar prediction accuracy, very close to that of the alignment-based distance measure. The prediction accuracy is not markedly affected by the length of *k*-mers from 3 to 6.

For clarity of presentation in the remaining of the paper, we let the *k*-mer size to be 6. Based on the sample size and distribution for each dataset, we choose *K* = 1 as the number of neighbors in KNN for the rabies virus dataset, and *K* = 7 for the coronavirus dataset and influenza A virus dataset. For alignment-free distance measures, we use Manhattan distance as an representative as many of them have similar prediction accuracies.

### Results based on the rabies virus dataset

**Figure 1:**
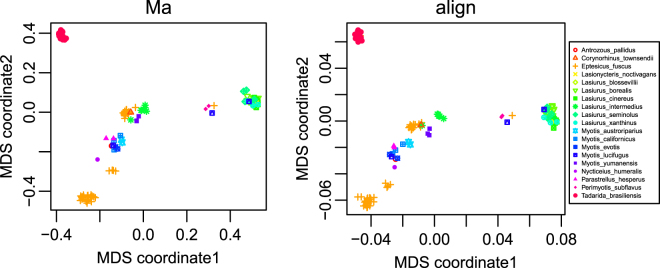
MDS plots for the 148 rabies viruses with complete N gene sequences based on the Manhattan distance using 6-mers (left) and alignment (right). Each point in the plots is a sample colored by the host species’ name.

**Figure 2:**
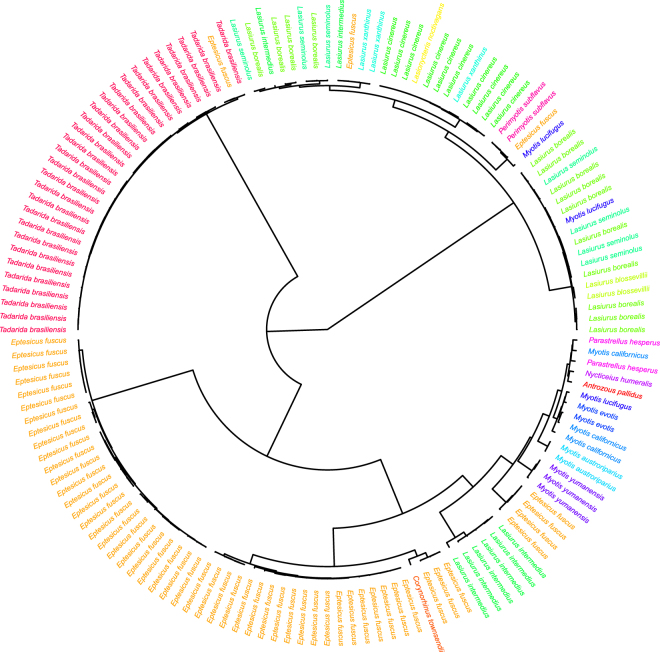
Hierarchical clustering of 148 rabies viruses with complete N gene sequences using the alignment-based distances of the virus sequences. Each leaf in the figure is a virus sample colored by the host species’ name.

**Figure 3:**
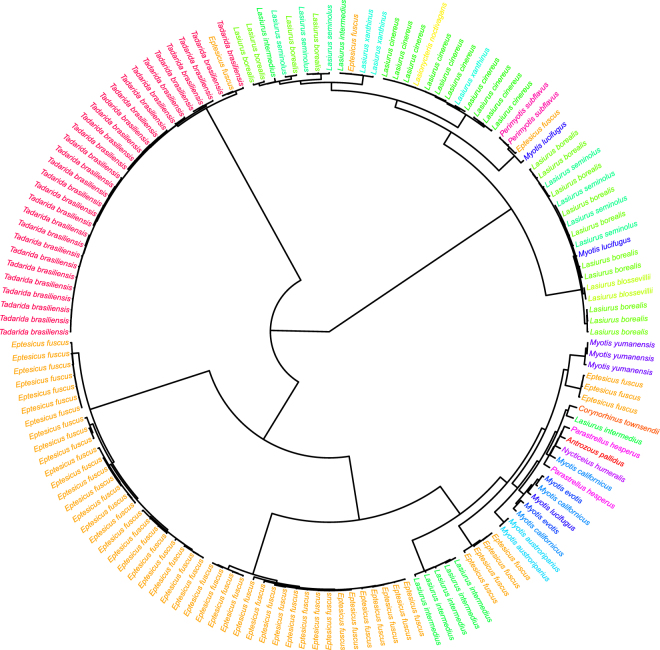
Hierarchical clustering of 148 rabies viruses with complete N gene sequences based on the Manhattan distance between the 6-mer frequencies of the virus sequences. Each leaf in the figure is a virus sample colored by the host species’ name.

Figure 1 shows the multidimensional scaling (MDS) plots of the 148 rabies viruses with complete Nucleoprotein (N) gene sequences based on the Manhattan distance using 6-mers (left) and alignment (right). In addition, Figs 2 and 3 show the hierarchical clustering of the viruses using alignment-based distance and Manhattan distances using 6-mers, respectively. The clustering results using the alignment-based method and the Manhattan distance are highly similar indicating that the alignment and alignment-free based methods give roughly similar results.

**Figure 4:**
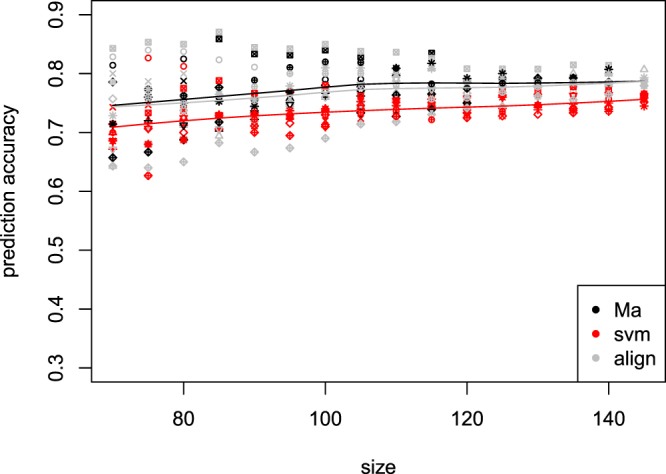
The prediction accuracy for different sample sizes for the rabies virus dataset using alignment-based distance, Manhattan distance with 6-mers and one nearest neighbor, and SVM. The smooth lines are the fitted curves for the mean prediction accuracy for different sample sizes. Ma: Manhattan distance; align: Alignment based method; SVM: support vector machine based method.

Figure 4 shows the leave-one-out cross-validation (LOOCV) prediction accuracies of one nearest neighbor using alignment-based distance and Manhattan distance with 6-mers, and SVM for different sample sizes. It can be seen from the figure that the average prediction accuracies for the alignment based method and the Manhattan distance based method are similar. However, the prediction accuracies for the alignment-based method have an relatively larger variance than that for the Manhattan distance based method for almost all the sample sizes considered. On the other hand, the average prediction accuracies for the SVM based method are lower than that for both the alignment and Manhattan distance based methods.

We also use 10-fold and 20-fold cross-validation to estimate the prediction accuracy and the results are given in Fig. S4 in supplementary material. The 10-fold and 20-fold cross-validation results also support that alignment based method and the Manhattan distance based method have highly similar performance. Comparing Fig. 4 with Fig. S4, we can see that the prediction accuracies increase with “N” for N-fold cross-validation, which is reasonable since the proportion for the training dataset increases with “N”.

### Results based on the coronavirus dataset

**Figure 5:**
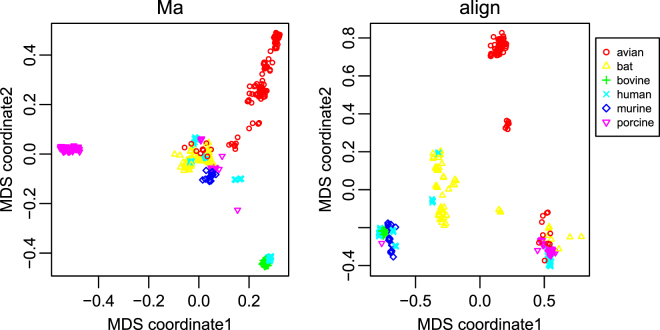
MDS plots for the 707 coronaviruses with spike gene sequences based on the distances calculated by the manhattan distance with 6-mers (left) and alignment (right). Each point in the plots is a sample colored by the host’s name.

**Figure 6:**
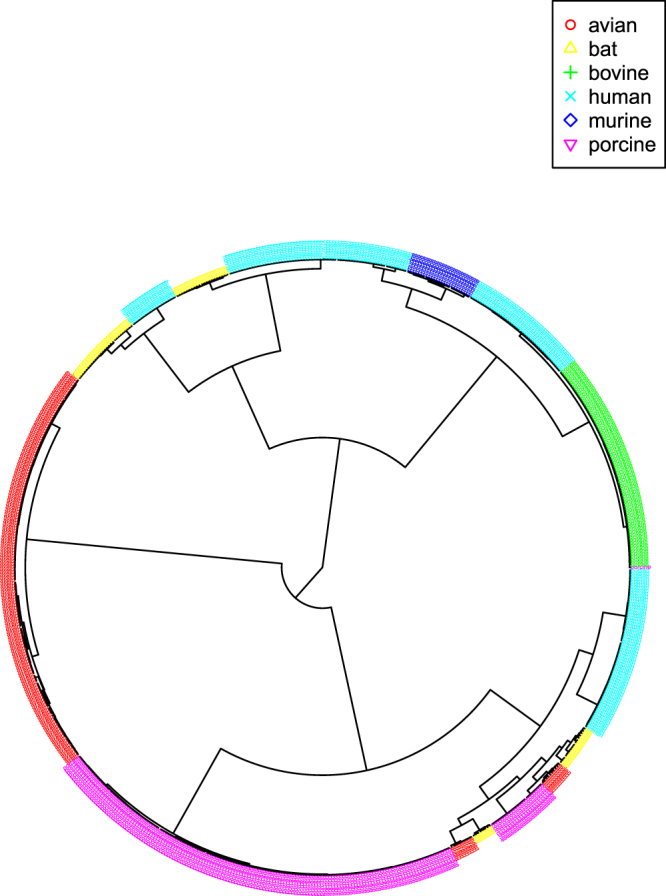
Hierarchical clustering of the 707 coronaviruses with spike gene sequences using the alignment-based distances of the virus sequences. Each leaf in the figure is a virus sample colored by the host’s name.

**Figure 7:**
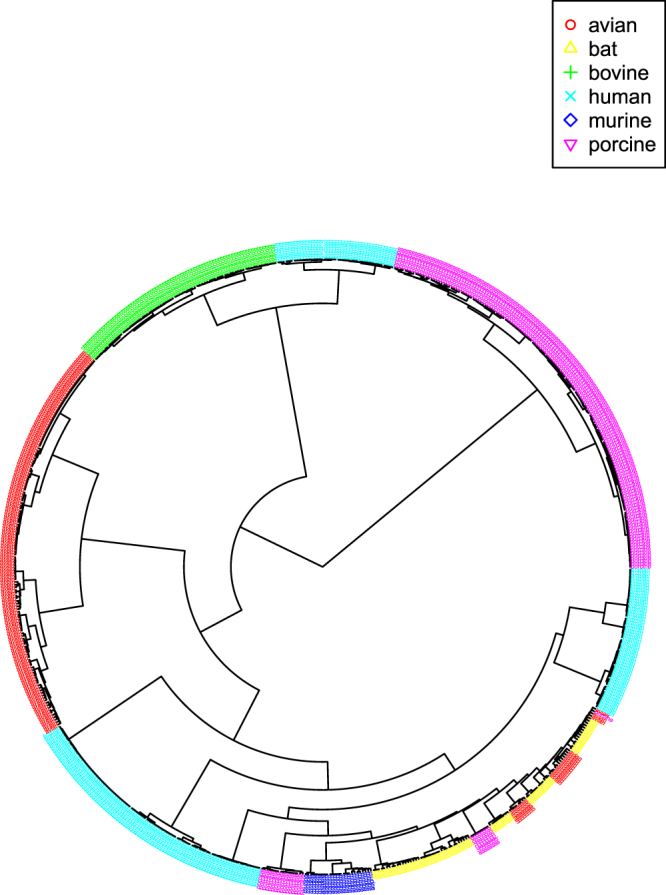
Hierarchical clustering of the 707 coronaviruses with spike gene sequences using the Manhattan distance between the 6-mer frequencies of the virus sequences. Each leaf in the figure is a virus sample colored by the host’s name.

**Figure 8:**
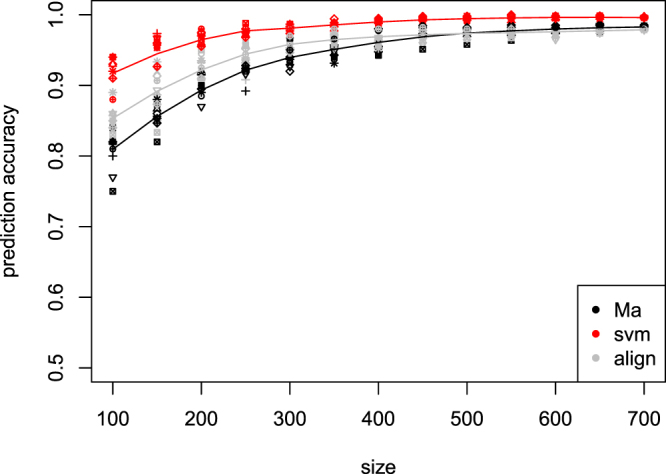
The prediction accuracy for different sample sizes for the coronaviruses dataset using alignment-based distance, Manhattan distance with 6-mers and 7 nearest neighbor, and SVM. The smooth lines are the fitted curves for the mean prediction accuracy for different sample sizes. Ma: Manhattan distance; align: Alignment based method; SVM: support vector machine based method.

Similar to the rabies virus dataset, we first visually check the pairwise distance matrix using MDS and hierarchical clustering. Figure 5 shows the MDS plots of the 707 coronaviruses with spike gene sequences based on alignment-free and alignment distances. Figures 6 and 7 show the hierarchical clustering of the viruses using alignment-based distance and the Manhattan distance with *k*-mer length of 6, respectively. Figure 8 shows the LOOCV prediction accuracies of the coronavirus dataset using alignment-based distance and Manhattan distance, and SVM for different sample sizes. The prediction accuracies for all the methods are greater than 0.90 when the sample size is above 400. When the sample size is <400, the prediction accuracy of SVM is somewhat higher than the KNN methods. Similar results are observed based on 10-fold and 20-fold cross-validations as shown in Fig. S5 in supplementary material.

### Results based on the influenza A dataset

**Figure 9:**
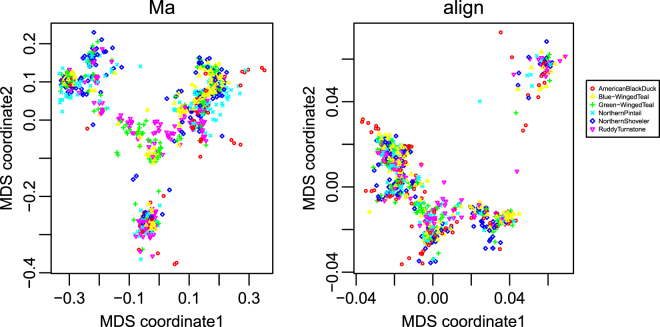
MDS plots for the influenza A viruses with N gene sequences based on the distances calculated using Manhattan distance of 6-mer frequencies (left) and alignment method (right). Each point in the plots is a sample colored by the host species’ name.

**Figure 10:**
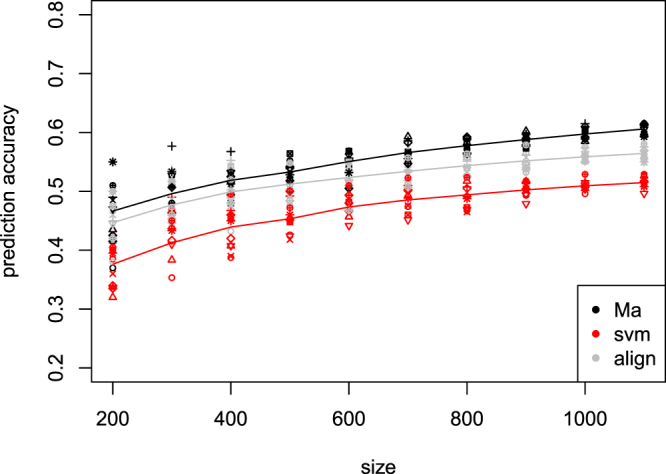
The prediction accuracy for different sample sizes for influenza A dataset using alignment-based distance, Manhattan distance with 6-mers and 7 nearest neighbor, and SVM. The smooth lines are the fitted curves for mean prediction accuracy for different sample sizes.

Parallel to the investigations for the rabies virus and the coronavirus, Fig. 9 shows the MDS plots for the 1,200 influenza A viruses with N gene sequences based on Manhattan distance using 6-mers and alignment method. Figures S6 and S7 show the corresponding hierarchical clustering results. The MDS and hierarchical clustering plots do not show a clear clustering pattern according to the hosts. A potential explanation is that the sources of the influenza A virus data are much more diverse and consist of several different virus clades. Figure 10 shows the LOOCV prediction accuracies of the influenza A virus dataset using alignment-based distance, Manhattan distance using 6-mers, and SVM for different sample sizes. The prediction accuracies for alignment and alignment-free method are similar, and are higher than that of SVM method for this dataset. Similar results are obtained based on 10-fold and 20-fold cross-validations as shown in Fig. S8 in supplementary material.

## Discussion

In this paper, we investigate the use of alignment-based and alignment-free distance methods and support vector machine to predict the host of viruses based on three virus datasets: rabies virus, coronavirus and influenza A virus. None of the three methods consistently outperforms other methods. For the rabies virus dataset, the alignment based and alignment-free methods perform similarly and both outperform SVM with a large margin. For the coronavirus dataset, SVM outperforms alignment based method followed by alignment-free method when the sample size is low. When the number of samples is large, eg. over 400, all the three methods perform similarly. Finally, for the influenza A virus, none of the methods performs well with prediction accuracies below 0.6. The alignment-free method does a little bit better than the alignment based method and both outperform SVM. Thus, this study shows both alignment-based and alignment-free methods can be effectively used to predict the hosts of viruses.

Figures S1–S3 in the supplementary material show that the prediction accuracies of the various alignment-free and alignment methods tend to have large variation for *K* from 1 to 5 and become stable for *K* from 5 to 10. Therefore, we choose *K* = 7 for the coronaviruses and influenza A viruses datasets, and choose *K* = 1 for the rabies viruses dataset, since the sample size of the rabies dataset is small and the viruses are unevenly distributed in different host species, while the sample sizes of the other two datasets are much larger.

For both the rabies and the influenza A viruses, we use the nucleoprotein gene sequences, while for the coronavirus we use the spike gene. It was shown before that the spike gene evolves fast to better prevent the virus from detection by the host^16^, while nucleoprotein gene is much more conserved, especially at the non-synonymous sites^17,18^. For conserved genes like nucleoprotein genes, longer *k*-mers than mono- and dinucleotides are needed to distinguish the sequences. Therefore, SVM using only mono- and dinucleotide does not perform as well as alignment based or Manhattan distance using 6-mers for the rabies and influenza A viruses. On the other hand, for highly divergent genes such as the spike genes, mono- and dinucleotide frequencies are enough to capture the differences among the sequences resulting in better performance of the SVM method.

Our study has several limitations. First, since viruses can jump from one host to another, a virus can belong to multiple hosts. We use the host which the virus is discovered from as its only host. However, the virus may also use other unknown species different from the one it was discovered from as hosts. This will influence the prediction accuracy for both alignment and alignment-free methods. Second, we only investigate three types of viruses and there are many other types of viruses available. More studies are needed to see the general applicability of our prediction methods.

In conclusion, our study shows that both alignment based and alignment-free methods can be successfully used to predict the hosts of viruses. Therefore, when alignment is difficult or too time-consuming, alignment-free methods provide a promising alternative to predict the hosts of new viruses.

## Materials and Methods

### Materials

We analyze three virus datasets with different characteristics: rabies, coronavirus, and influenza A virus, to see if consistent results related to the relative performance of alignment, alignment-free, and machine learning based approaches can be obtained.

The rabies virus dataset from Streicker *et al*.^5^. The rabies virus is a single-stranded RNA virus and has a wide host range. We first investigate the rabies virus dataset from Streicker *et al*.^5^ consisting of 372 rabies virus samples from 23 bat host species. Among them, 148 viruses have complete N gene (1,353 bp) sequenced. In this paper, we concentrate on the study of these 148 viruses. The accession numbers of the complete genomes and N gene of the viruses were provided in Streicker *et al*.^5^ and the corresponding gene sequences can be downloaded from NCBI genbank database using their accession numbers at https://www.ncbi.nlm.nih.gov/genbank/.

The coronavirus dataset from Tang *et al*.^10^. Tang *et al*.^10^ developed a SVM based method using mono- and di-nucleotide sequences to predict the host of coronavirus. We use the same data as in Tang *et al*.^10^ consisting of 724 coronavirus samples from 6 host species (human, porcine, bovine, bat, murine and avian). Among them, 392 samples have complete genome sequenced, and 326 samples only have their spike genes sequenced. We extract the spike gene sequences from the complete genomes by checking the coding sequence annotation in NCBI and obtain additional 381 extracted spike gene sequences. Together with the original 326 sequences, we have a total of 707 all spike sequences and we focus on the investigation of these 707 spike sequences.

Influenza A virus dataset from the Influenza Research Database^19^. Finally, we investigate the host of influenza A virus as in Kargarfard *et al*.^11^. We collect the avian influenza A virus from the Influenza Research Database^19^ and exclude those sequences with ambiguous host species such as chicken, duck, avian, and gull, and also those host species with less than 200 virus sequences in the database. We restrict the samples to the same taxonomic rank, and choose the level as “species” in the taxonomic hierarchy. The prediction can surely be easier for more general categories. There are six remaining avian host species: American Black Duck *Anas rubripes*, Blue-Winged Teal *Anas discors*, Green-Winged Teal *Anas carolinensis*, Northern Pintail *Anas acuta*, Northern Shoveler *Anas clypeata*, and Ruddy Turnstone *Arenaria interpres*, for further study. For each host species, we randomly choose 200 virus sequences in our study.

### Computational methods

#### Calculate the pairwise distance/dissimilarity matrix of viruses

We compare the performance of alignment-based, alignment-free, and machine learning based approaches for inferring the hosts of viruses. For the alignment-based method, we first use the software “Clustal Omega”^20^ for multiple sequence alignment using the default parameters and then use the software “Phylip”^21^ and choose the “F84” evolutionary model to calculate the pairwise distance using the alignment results as input. We also investigate several alignment-free methods for calculating the distances/dissimilarities between viral sequences using the CAFE package^15^ and these include Chebyshev, Euclidean, Manhattan, CVTree^22^, *d*_2_, ${d}_{2}^{\ast }$, ${d}_{2}^{S}$ ^13^, etc. The definitions of these distances/dissimilarities were given in Lu *et al*.^15^. Our objective is to evaluate if alignment-free approaches have similar accuracies in predicting the hosts of virus sequences, but with much high computational efficiency.

#### Visualize the distance/dissimilarity matrix

To empirically see if viruses from the same host tend to be more similar to each other than those from different hosts, we first use MDS^23^ to project the virus sequences onto two-dimensional Euclidean space. MDS is a non-linear dimensionality reduction method that can reduce the pairwise distance matrix to lower dimensional space, while best recapitulating the original distance matrix. We also use hierarchical clustering with average linkage to visualize the relationship among the viruses, and intuitively assess whether the viruses infecting the same hosts are indeed closer than those infecting different hosts.

#### Predict the host of a virus

We apply KNN^24^ method based on the pairwise distance matrix for both alignment-based and alignment-free distances for predicting the host of the virus. The alignment-free distance measures include Chebyshev (Ch), Euclidean (Eu), Manhattan (Ma), CVTree (CVT), *d*_2_, ${d}_{2}^{\ast }$ and ${d}_{2}^{S}$ with various *k*-mer sizes. For each virus, we choose the *K* viruses that are closest to the virus from the pairwise distance matrix, and then count the frequency of the hosts of the *K* viruses. We use the most frequent host as the predicted host of the virus. For machine learning based prediction, we use SVM based on mono– and dinucleotide frequencies (3 mononucleotide frequencies and 16 dinucleotide biases^10^). R package e1071 was used for SVM analysis with “C-classification” as the model type and “Radial” as the SVM kernel^10^.

We use LOOCV^25^ and *N*-fold cross-validation to evaluate the prediction accuracy. We implement this process for all the viruses and then compare the predicted host with its true host to obtain the prediction accuracy.

#### Investigate the impact of sample size on prediction accuracy

The number of known sequences for each host can significantly affect the prediction accuracy. In order to quantify the effects of sample size on prediction accuracy, we randomly choose a certain number of sequences and then apply the KNN and SVM approaches to the set of sequences to obtain the prediction accuracy as described above. We repeat this process for a series of sample sizes to see how the prediction accuracy changes with sample size. We let the sample size change from 70 to 145 with a step size of 5 for the rabies virus dataset, from 100 to 700 with a step size of 50 for the coronavirus, and from 200 to 1100 with a step size of 100 for the influenza A virus dataset. For each sample size, we randomly choose 10 sets of sequences and calculate the prediction accuracy for each dataset.

### Data availability

All data are publicly available online and can be found based on the information provided in Materials and Methods part.

## Electronic supplementary material

Supplementary information
