# Supplementary material for: Comparative studies of alignment, alignment-free and SVM based approaches for predicting the hosts of viruses based on viral sequences
Source: Sci Rep. 2018 Jul 3;8:10032. doi: 10.1038/s41598-018-28308-x (PMC6030160; doi:10.1038/s41598-018-28308-x)
Supplement: Supplementary file 1 — Supplementary information [file 41598_2018_28308_MOESM1_ESM.pdf]

Supporting Information

Comparative studies of alignment, alignment-free and SVM based approaches for predicting the hosts of viruses based on viral sequences

Han Li[1], Fengzhu Sun[1,2*]

[1]Molecular and Computational Biology Program, Department of Biological Sciences, University of Southern California, Los Angeles, CA 90089, USA.
[2] Centre for Computational Systems Biology, School of Mathematical Sciences, Fudan University, Shanghai 200433, China. Correspondence and requests for materials should be addressed to F.S. (email: fsun@usc.edu)

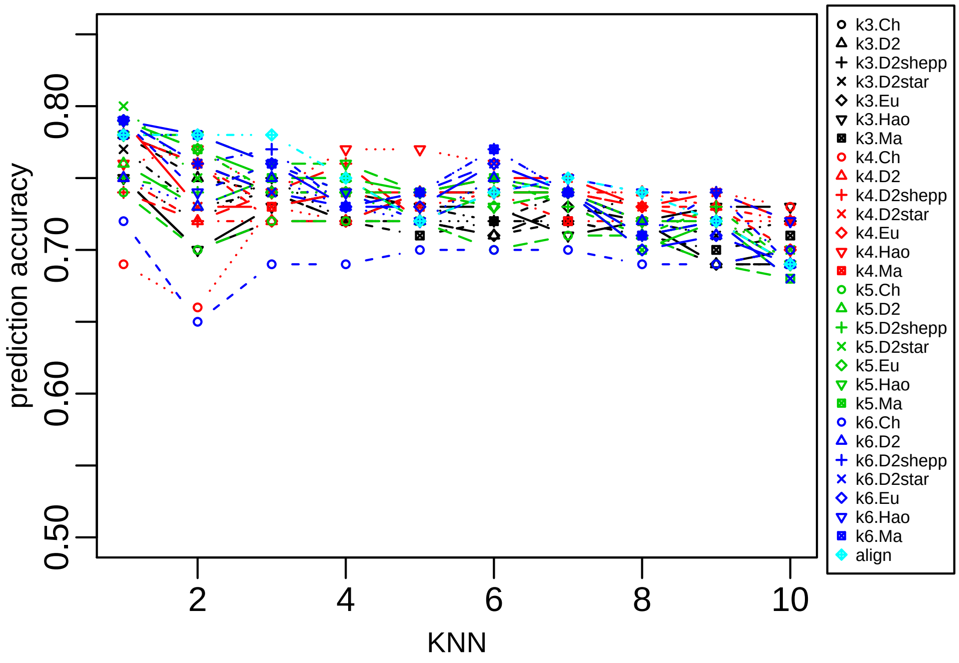

Figure S1: The prediction accuracy of the KNN algorithm for the rabies virus dataset using varying distance measures, k-mer length from 3 to 6, and the number of neighbors (K, horizontal axis).

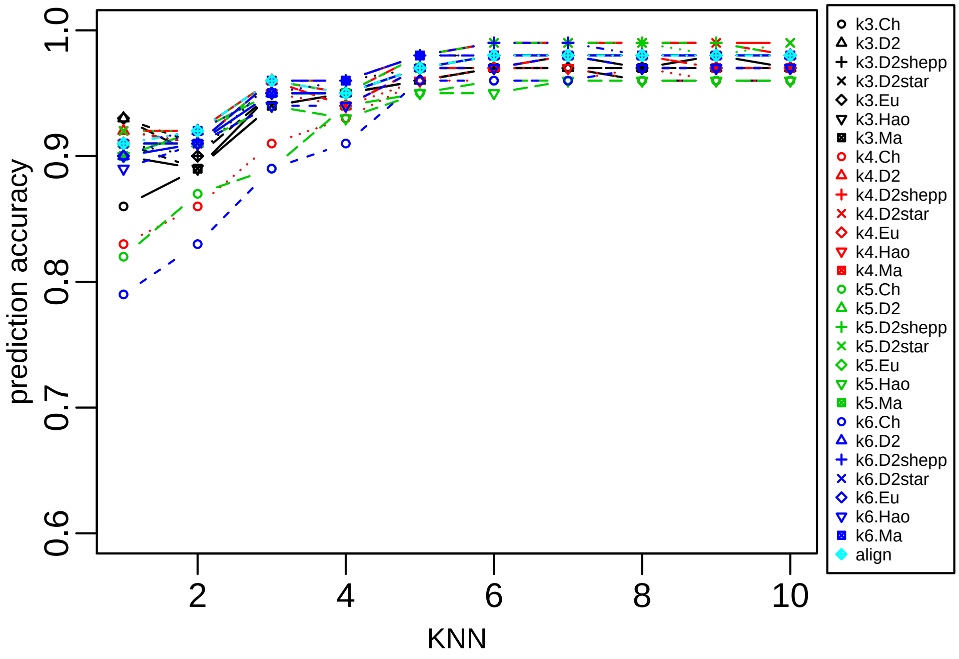

Figure S2: The prediction accuracy of the KNN algorithm for the coronavirus dataset using varying distance measures, k-mer length from 3 to 6, and the number of neighbors (K, horizontal axis).

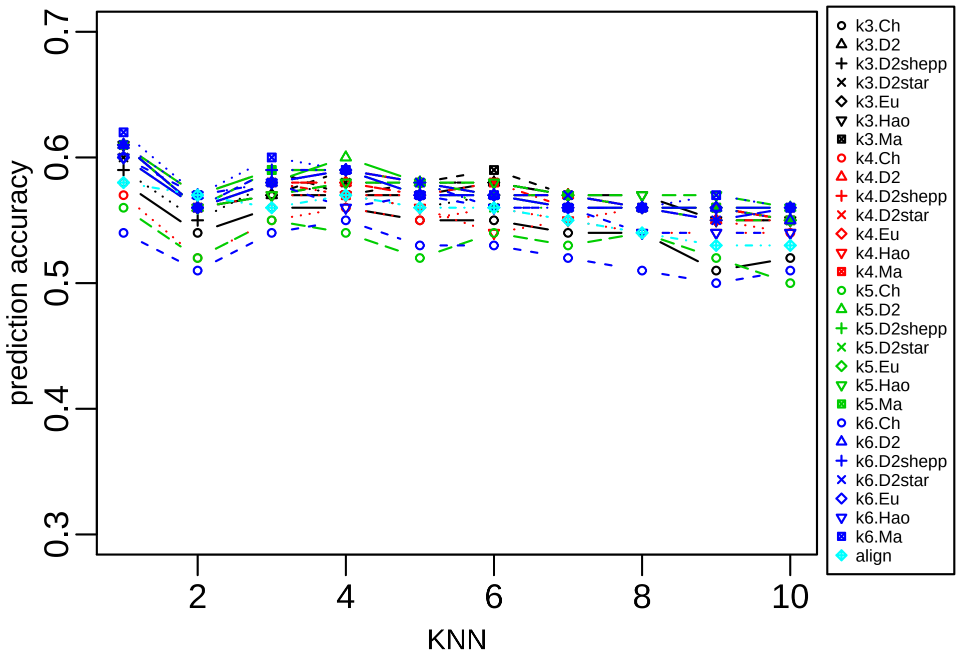

Figure S3: The prediction accuracy of the KNN algorithm for the influenza A virus dataset using varying distance measures, k-mer length from 3 to 6, and the number of neighbors (K, horizontal axis).

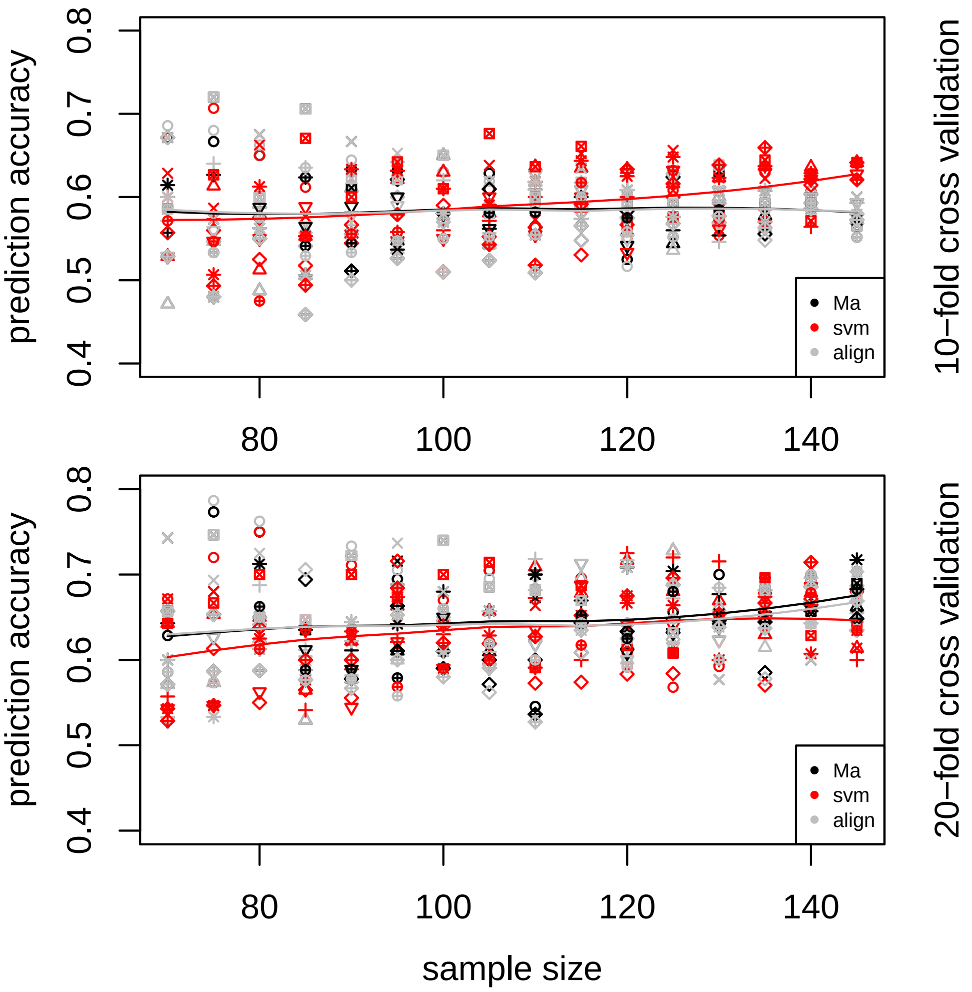

Figure S4: The prediction accuracy for different sample sizes for the rabies virus dataset using alignment-based distance, Manhattan distance with 6-mers and one nearest neighbor, and SVM using 10-fold (upper) and 20-fold (lower) cross-validation. The smooth lines are the fitted curves for the mean prediction accuracy for different sample sizes. Ma: Manhattan distance; align: Alignment based method; SVM: support vector machine based method.

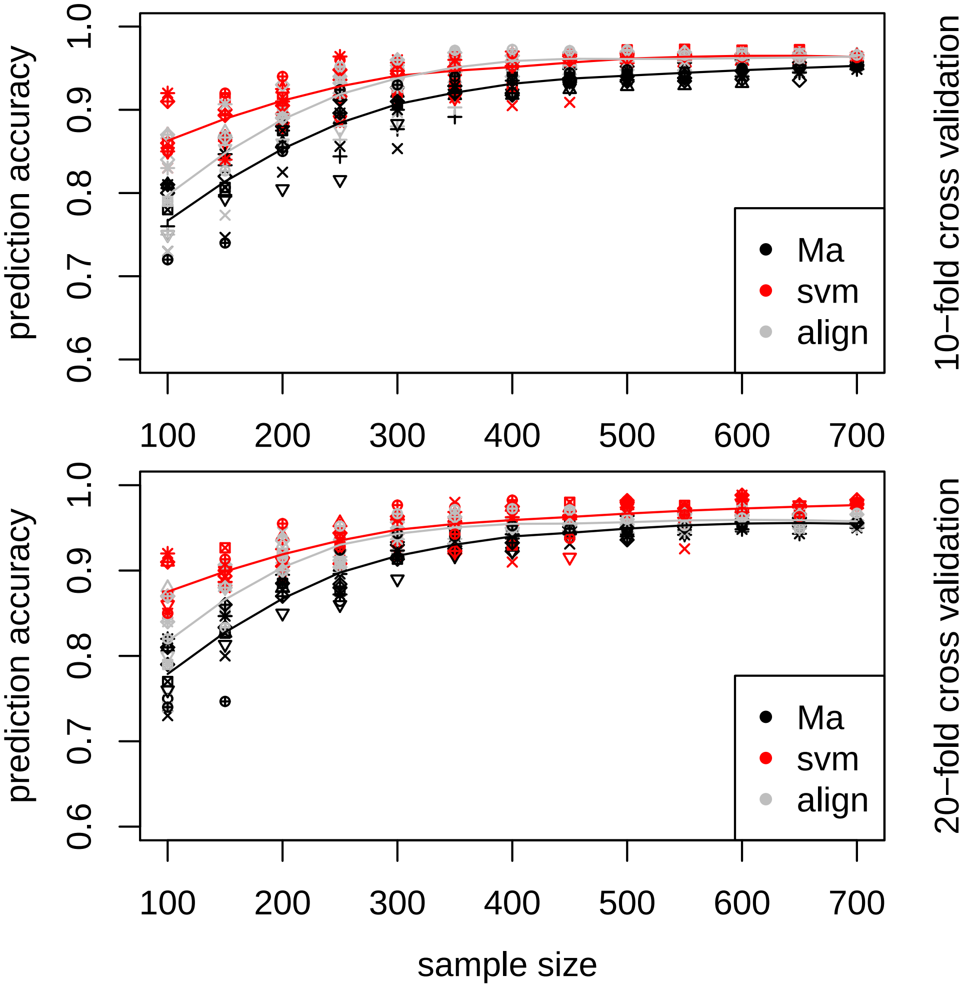

Figure S5: The prediction accuracy for different sample sizes for the coronavirus dataset using alignment-based distance, Manhattan distance with 6-mers and one nearest neighbor, and SVM using 10-fold (upper) and 20-fold (lower) cross-validation. The smooth lines are the fitted curves for the mean prediction accuracy for different sample sizes. Ma: Manhattan distance; align: Alignment based method; SVM: support vector machine based method.

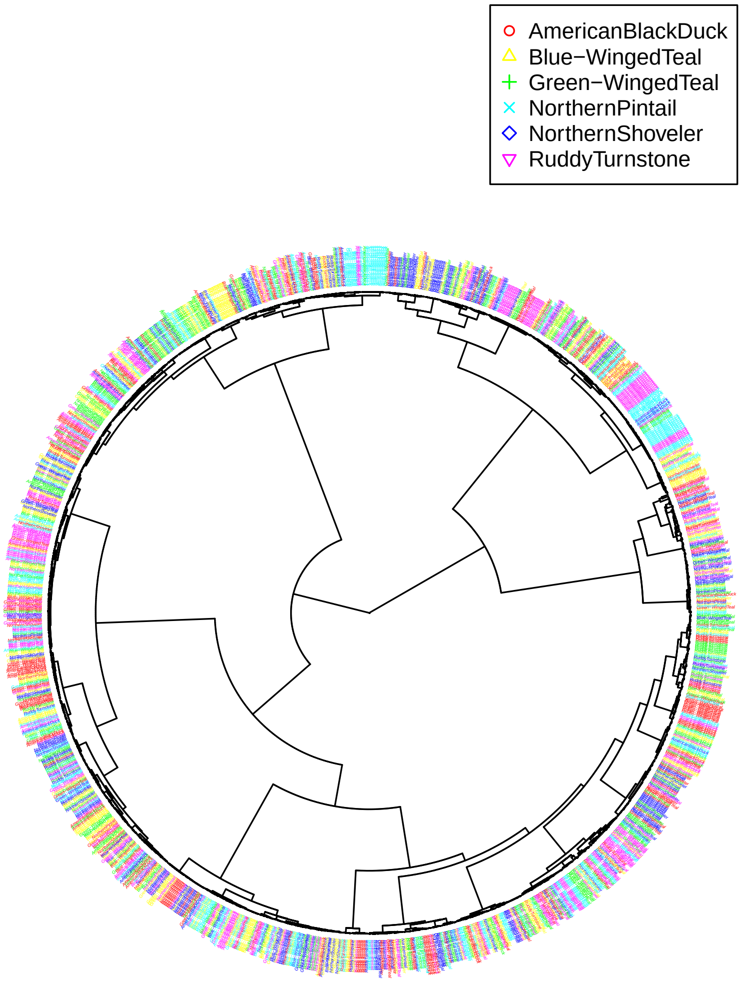

Figure S6: Hierarchical clustering of the influenza A viruses with N gene sequences using the alignment-based distances of the virus sequences. Each leaf in the figure is a virus sample colored by the host species' name.

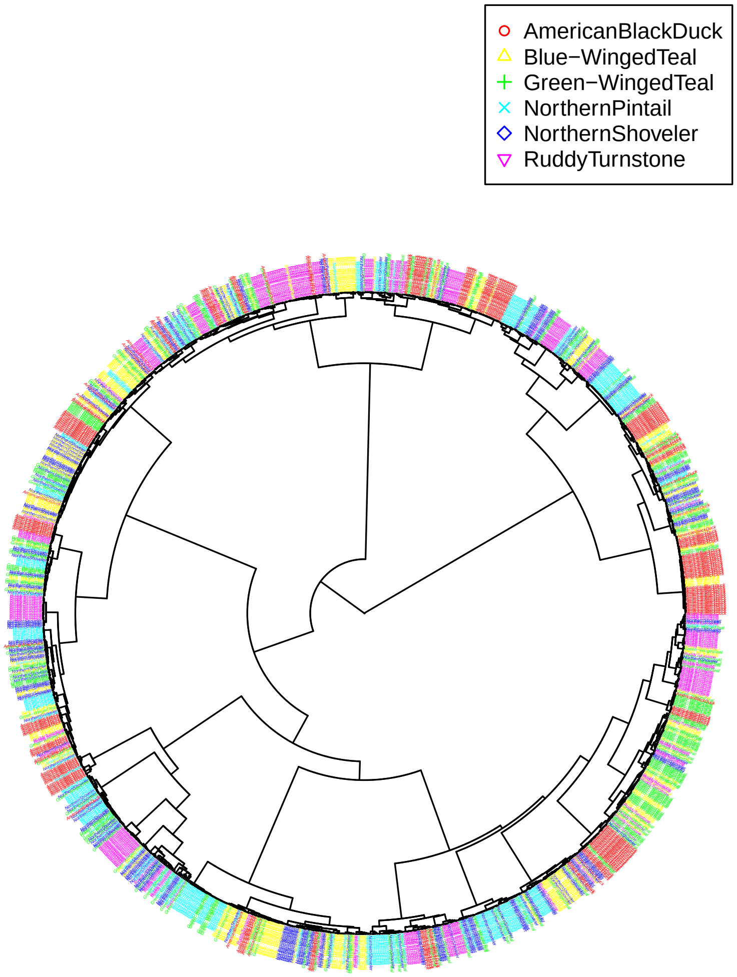

Figure S7: Hierarchical clustering of the influenza A viruses with N gene sequences using the Manhattan distance between the 6mer frequencies of the virus sequences. Each leaf in the figure is a virus sample colored by the host species' name.

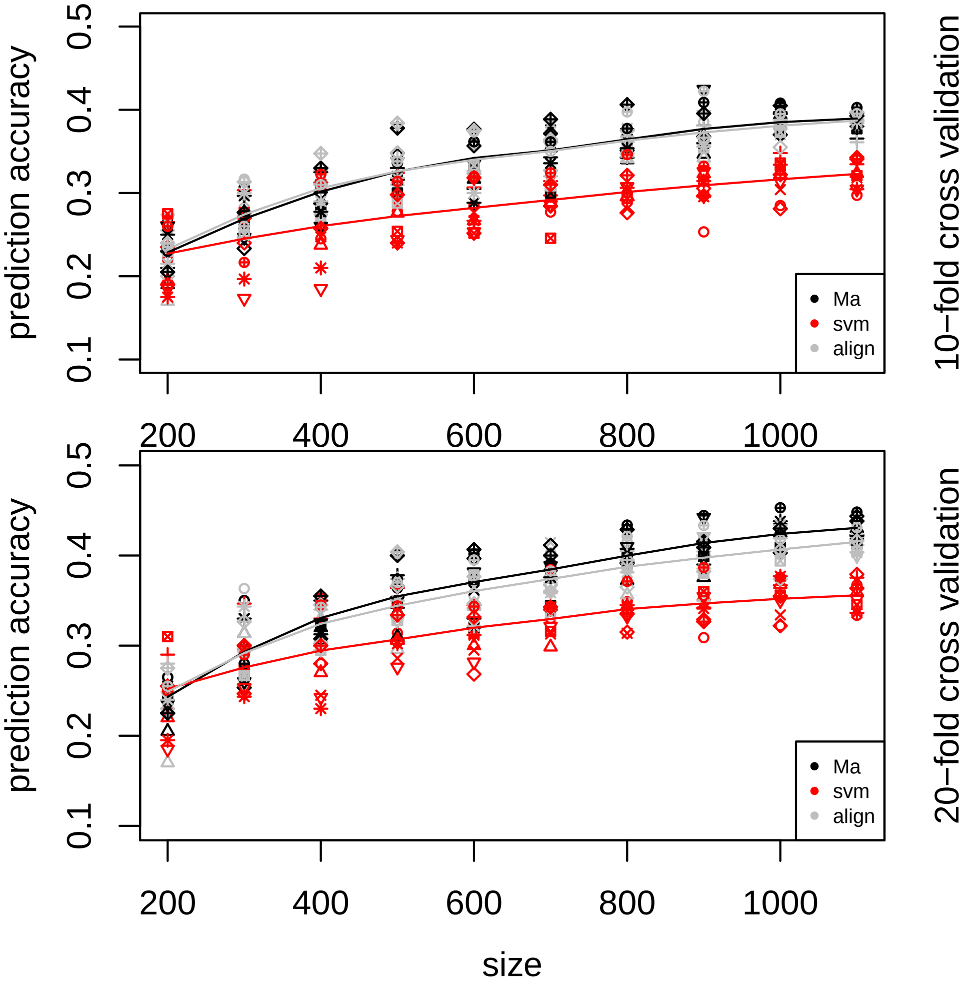

Figure S8: The prediction accuracy for different sample sizes for the influenza A virus dataset using alignment-based distance, Manhattan distance with 6-mers and one nearest neighbor, and SVM using 10-fold (upper) and 20-fold (lower) cross-validation. The smooth lines are the fitted curves for the mean prediction accuracy for different sample sizes. Ma: Manhattan distance; align: Alignment based method; SVM: support vector machine based method.
